# Collective Behavior of Market Participants during Abrupt Stock Price Changes

**DOI:** 10.1371/journal.pone.0160152

**Published:** 2016-08-11

**Authors:** Jun-ichi Maskawa

**Affiliations:** Department of Economics, Seijo University, Tokyo, Japan; East China University of Science and Technology, CHINA

## Abstract

Under uncertainty, human and animal collectives often respond stochastically to events they encounter. Human or animal individuals behave depending on others’ actions, and sometimes follow choices that are sub-optimal for individuals. Such mimetic behaviors are enhanced during emergencies, creating collective behavior of a group. A stock market that is about to crash, as markets did immediately after the Lehman Brothers bankruptcy, provides illustrative examples of such behaviors. We provide empirical evidence proving the existence of collective behavior among stock market participants in emergent situations. We investigated the resolution of extreme supply-and-demand order imbalances by increased balancing counter orders: buy and sell orders for excess supply and demand respectively, during times of price adjustment, so-called special quotes on the Tokyo Stock Exchange. Counter orders increase positively depending on the quantity of revealed counter orders: the accumulated orders in the book until then. Statistics of the coming counter order are well described using a logistic regression model with the ratio of revealed orders until then to the finally revealed orders as the explanatory variable. Results given here show that the market participants make Bayesian estimations of optimal choices to ascertain whether to order using information about orders of other participants.

## Introduction

When choosing from among several options or when evaluating something, if one lacks accurate information about circumstances, then one cannot make decisions with confidence. Under such uncertainty, probabilistic responses to issues that one faces are naturally expected: the proportion of people who choose A is *x*; the rest (1-*x*) choose B. Some people might make probabilistic estimation of optimal choices based on their own information. Others might blindly follow the actions that others take because of the difficulty of obtaining related information independently, especially during times of crisis.

A stock market that is about to crash or which is in the middle of an abrupt price change is an example of such a situation. For example, immediately after the Lehman Brothers bankruptcy, the world economy was unstable. Stock markets throughout the world were anticipating an impending large decline at any moment. No market participant had perfect information about market-related circumstances. Therefore, no one could know exactly how much or when stock prices would decline. Market participants who are facing such an urgent situation and who have insufficient time to make sufficient investigations about the circumstances independently will use the order placements of other participants as important additional information. A tendency for such mutual reference by market participants will elicit collective behavior among market participants. This study was conducted to provide empirical evidence and to describe such collective behavior of market participants under uncertain and emergent circumstances.

Studies of animal behavior have demonstrated that animals make probabilistic estimations of optimal choices under various uncertain ecological situations such as foraging in patches, choosing a mate, and avoiding predation risk [1]. Animals use the behavior of other animals as well as their own experiences as additional information to make probabilistic estimations about situations [2–5]. A well-designed experiment using a shoaling fish species demonstrated how their probabilistic decision-making depends on the behavior of other group members [3–5]. Experimental studies of decision-making under uncertainty about the group of human beings must also clarify the emergence of collective behavior among human subjects [6–8]. For fish and human cases, the Bayesian decision-making model has well described their collective behavior [3–5, 8].

In stock market studies, various theoretical works have been conducted. An important theoretical framework of the information-based collective behavior of market participants has been that of information cascades [9–12]. When the choice of an individual following the choice of the preceding individual without regarding his own information is optimal, the sequence made by the respective choices of individuals is called an information cascade [10]. Other important theoretical frameworks which are not fully rational as well as reports of empirical studies conducted up to 2000 have been reviewed in an IMF report by Bikhchandani and Sharma [13]. Recent agent-based approaches are important to connect the behavior of market participants to financial price movements [14–22].

Empirical evidence related to herding among players in financial markets abounds: the mutual influence on the recommendations of security analysts [23], momentum investment strategies taken by most mutual fund managers [24], bias (overoptimism), and herding of earning forecasts by financial analysts [25]. Many other studies of the herding in stock markets have emphasized collective price movements such as the cross-sectional standard deviation of returns [26], drawdowns (loss from the last local maximum to the next local minimum)[27], and correlations among stock returns [28, 29] because the trading bias created by the herding behavior of market participants is regarded as engendering wild price movements in all stocks. Actually, market-wide collective behavior of stock prices was observed before almost every crash that has occurred during the last decade [30, 31].

As described in this paper, to provide empirical evidence proving the existence of collective behavior among stock market participants, the author investigates processes of getting extreme supply-and-demand order imbalance out of the order book during the special quote, which is a special rule of the Tokyo Stock Exchange for price adjustment and which is described in some detail in the next section. We analyze the order flow during special quotes on 21 days of crashes and on 11 days of rebounds with amplitudes of daily returns greater than 5% that occurred at the Tokyo Stock Exchange (TSE) during the last decade. Results show that the coming orders with each passing time of the special quotes are well fitted to a logistic regression model with the accumulated orders up to then as the explanatory variable. The author also applies some alternative models for comparison. As discussed later, results explained herein can be interpreted in the framework of Bayesian probability estimation used in an analysis of experiments conducted with a group of fish [3–5] or a group of humans [8]. Results show that market participants use the order placement information of other participants to decide whether to order at a given time.

## Data and Analysis

### Transaction Rules of Stocks and Special quotes

#### Transaction Rules of Stocks

Main stock markets throughout the world share transaction rules. Each transaction session of the day starts by the opening batch auction in which all buy and sell orders accumulated up to that time are executed collectively at a price that maximizes the executed orders, followed by a continuous auction in which incoming orders are executed successively. The TSE has two sessions each day: morning and afternoon sessions with one hour intermission between. The TSE takes orders of two types: market orders and limit orders. Market orders are orders by market participants who want to buy or sell a specified quantity at any available price. In contrast, limit buy (sell) orders are orders for which the quantity and the maximum (minimum) bidding (asking) execution price are specified. In batch and continuous auctions, buy and sell orders are executed according to the price and the time priority rule. Market orders are executed by top priority.

#### Special quote

For updates of stock prices, the TSE has a special rule that does not exist in other main stock markets throughout the world: not in New York, Nasdaq, London, Paris, Frankfurt, Hong Kong, or other exchanges. On the TSE, the next execution price cannot jump beyond the given price interval defined depending on the price range of the previous execution price, thereby preventing wild short-term price fluctuations. A circuit breaker is another rule, used in other markets, that stops trading for a period of time to cool down the market when an abrupt drop of stock price took place.

When the next execution might take place at a price beyond these price intervals because of a supply demand order imbalance, the TSE indicates a special quote and the transaction is interrupted. A special quote notifies market participants that there is an order imbalance at the quote price *Q*(*t*_0_), which is the highest or lowest executable price according to the rule, i.e., *Q*(*t*_0_) = *P*(*t*_0_) ± Δ*p*. The quote encourages participants to place balancing orders on the other side of the order book, where *P*(*t*_0_) and Δ*p* respectively denote the last execution price until time *t*_0_ and the maximum renewal price interval. If sufficient counter orders are placed to remove the order imbalance, then the transaction resumes. If orders are insufficient, then the special quote price will be renewed at a fixed time interval, i.e., *Q*(*t*_*i*+1_) = *Q*(*t*_*i*_) ± Δ*p* (*i* = 0, 1, 2, 3, …), until equilibrium is regained. The special quote price is renewed every 3 min now. According to the rule implemented until 9 May 2011, the special quote price was renewed every 5 min. Regarding trading rules and details of the special quotes, see the TSE website: http://www.jpx.co.jp/english/equities/trading/domestic/03.html.

**Fig 1:**
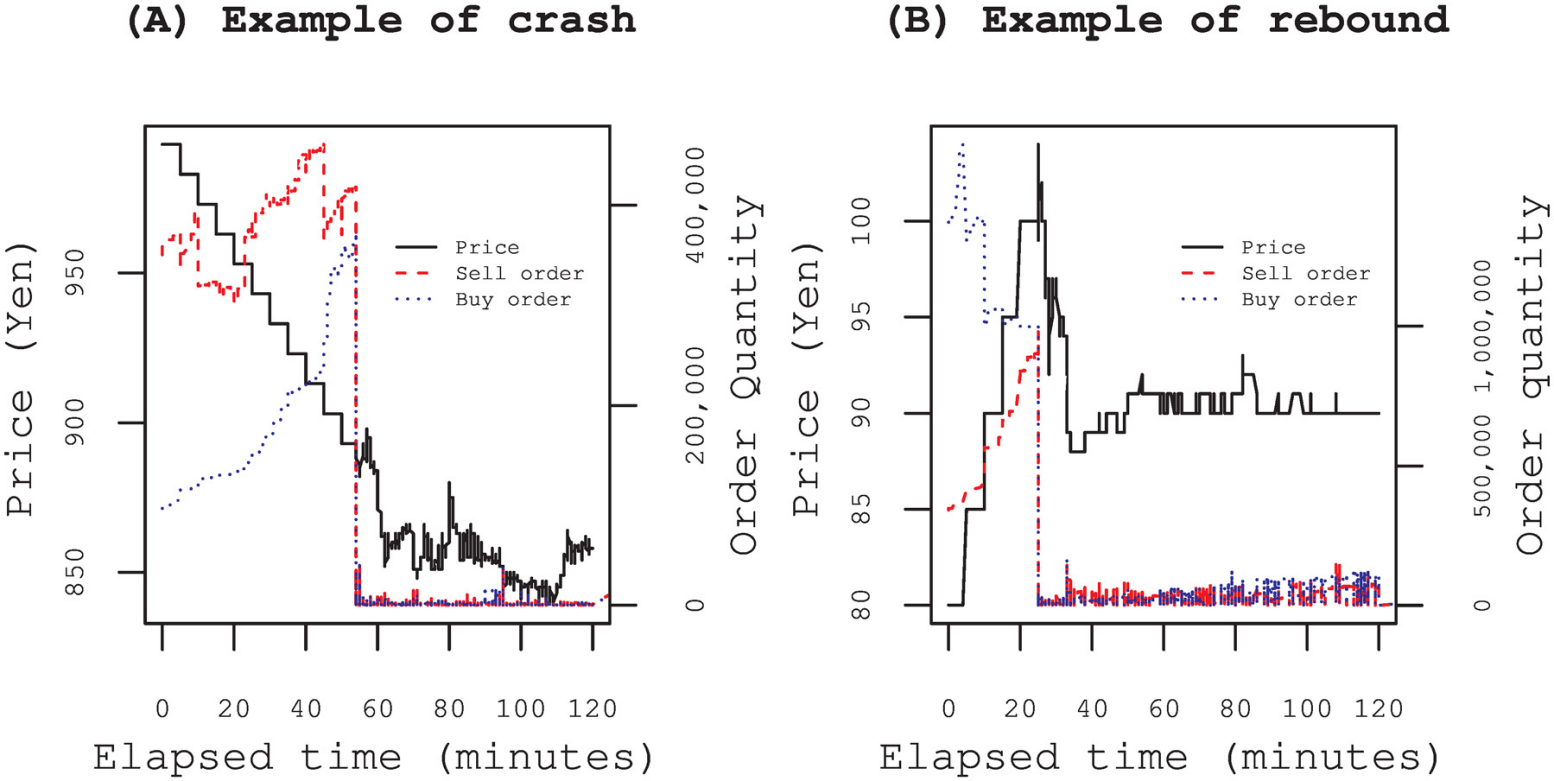
Examples of abrupt changes of stock prices during special quotes. Each Panel shows the price change and the order quantities of sell and buy sides during the two-hour morning session including the period of the special quote. (A) Example from the market crash of 16th Oct. 2008. The issue is Toyota Tsusho Corp. (Trading Co.). The equilibrium of sell and buy orders at the opening of the morning session was far below the last execution price of 1013 yen of the prior day. A special quote was indicated with the initial price of 993 yen, which is just a given renewal price interval 10 yen below the last price and the price was renewed at 5 min intervals. Although there is a large imbalance between sell and buy orders at the initial price, it had gradually blown over because of the increase of buy orders over time. The special quote had continued for 54 min until order supply and demand reached the equilibrium and ended with the price of 893 yen. (B) Example from the market rebound on 14 Oct. 2008. The issue is Oki Electric Ind. Co. Ltd. (Electric Machinery). The equilibrium of sell and buy order at the opening of the morning session was far above the last execution price 75 yen of the prior day. A special quote was indicated with the initial price of 80 yen, which is just a given renewal price interval 5 yen above the last price. The price had been renewed at 5 min intervals. The special quote had continued for 25 min until order supply and demand reached the equilibrium and ended with the price of 104 yen.

In Fig 1, two examples showing the evolution of the quote price and buy and sell orders during the morning session are shown, including the period of special quotes indicated immediately after the opening of the session. One is an example from the market crash on 16 Oct. 2008. The other is an example from the market rebound on 14 Oct. 2008. Both stocks experienced abrupt price changes in an extremely short time. Market participants placing counter orders can buy at a lower price or sell at a higher price by deferring an order at the risk of losing an opportunity to trade because the special quote would be closed by the counter orders of other market participants who believe that buying or selling at the quoted price is the optimal choice. The final price determined by the closing time of the special quote is uncertain for market participants. Therefore, they should decide when to place orders under such uncertain circumstances.

### Data

We investigate the order flow during the time when the Tokyo stock Exchange (TSE) has indicated special quotes on days when a sharp decline or increase of the daily log-return defined by the logarithm of the ratio of the closing price of the day to the closing price of the prior day of the Nikkei 225 Index had amplitude greater than 5%. In the past decade, 21 days have had such declines of less than -5% and 11 days of increases greater than 5%.

Those are shown in Tables 1 and 2 in descending order of the amplitude of the daily log-return for the price-decline and the price-increase days, respectively. The target issues of our empirical analysis are the constituent issues of the Nikkei 225 Index at the moment of September 2015, which is shown in Table in S1 Table, and listed on the TSE on such days of crashes and rebounds investigated here.

**Table 1:** Daily log-returns of the Nikkei Index less than -5% during the past decade.

Rank	Date	Daily ret. of Nikkei 225	# of s. q./# of target issues	Avg. ret.	Avg. ret. during s. q. (Std.)
1	10/16/2008	-12.1%	211 / 215	-12.4%	-10.1% (3.1%)
2	3/15/2011	-11.2%	174 / 222	-12.8%	-9.0% (4.6%)
3	10/10/2008	-10.1%	215 / 215	-9.1%	-12.8% (3.8%)
4	10/24/2008	-10.1%	86 / 215	-11.0%	-4.8% (2.5%)
5	10/8/2008	-9.9%	182 / 215	-10.5%	-4.1% (1.5%)
6	5/23/2013	-7.6%	8 / 224	-12.5%	-3.0% (0.7%)
7	11/20/2008	-7.1%	163 / 215	-8.1%	-4.6% (2.5%)
8	10/22/2008	-7.0%	132 / 215	-8.8%	-4.0% (1.5%)
9	11/6/2008	-6.8%	196 / 215	-7.3%	-5.5% (2.5%)
10	10/27/2008	-6.6%	75 / 215	-11.5%	-2.7% (2.2%)
11	12/2/2008	-6.6%	196 / 215	-7.1%	-6.0% (2.0%)
12	6/13/2013	-6.6%	106 / 224	-6.6%	-2.9% (0.6%)
13	3/14/2011	-6.4%	205 / 222	-8.3%	-8.8% (4.8%)
14	1/22/2008	-5.8%	178 / 215	-6.5%	-4.2% (1.6%)
15	12/12/2008	-5.7%	161 / 215	-6.5%	-4.0% (1.6%)
16	8/17/2007	-5.6%	74 / 214	-9.0%	-2.8% (1.1%)
17	11/13/2008	-5.4%	179 / 215	-5.8%	-5.1% (1.7%)
18	5/30/2013	-5.3%	94 / 224	-5.2%	-3.3% (0.9%)
19	10/31/2008	-5.1%	105 / 215	-7.5%	-5.0% (3.1%)
20	9/16/2008	-5.1%	200 / 215	-5.3%	-6.0% (3.2%)
21	1/15/2009	-5.0%	186 / 218	-4.7%	-4.1% (1.5%)

Rank of the amplitude of decline, date, and daily log-return are shown in the first three columns. The number of issues for which the special quote has been indicated among target issues of the day and the average daily log-returns of those issues are shown respectively in the fourth and the fifth columns. The last column shows the average log-returns of those issues during the special quote and the standard deviation in parentheses.

**Table 2:** Daily log-returns of the Nikkei Index larger than 5% for the past decade.

Rank	Date	Daily ret. of Nikkei 225	# of s. q./# of target issues	Avg. ret.	Avg. ret. during s. q. (Std.)
1	10/14/2008	13.2%	215 / 215	14.6%	13.5% (5.0%)
2	10/30/2008	9.5%	96 / 215	11.8%	5.2% (2.9%)
3	10/29/2008	7.5%	182 / 215	7.2%	8.1% (3.6%)
4	10/28/2008	6.2%	41 / 215	9.5%	3.2% (1.6%)
5	11/4/2008	6.1%	174 / 215	6.9%	5.4% (2.6%)
6	11/10/2008	5.6%	190 / 215	5.9%	5.3% (2.7%)
7	3/16/2011	5.5%	144 / 222	7.7%	6.4% (4.0%)
8	11/25/2008	5.1%	201 / 215	4.7%	5.9% (2.4%)
9	12/15/2008	5.1%	160 / 215	5.6%	4.0% (1.7%)
10	12/8/2008	5.1%	52 / 215	6.4%	3.0% (1.4%)
11	3/13/2009	5.0%	145 / 218	5.0%	4.0% (1.7%)

The quantity shown in each column is the same as that in Table 1.

In the fourth columns of Tables 1 and 2, we present the number of issues for which the TSE indicated the special quote because of the large imbalance between the demand and the supply orders. In those tables, we also show the average daily log-returns of issues on which the special quotes have been indicated and the average log-returns of those issues during the special quote. The special quote has been indicate on the total 3126 (1600) issues, which amount to 67% (67%) of 4698 (2375) target issues, and the log-return during the special quote accounts for 74% (84%) of the total daily log-return of those issues in the price-decline (price-increase) days. Those data demonstrate that the special quote has accounted for a substantial fraction of the price movements during crashes and rebounds that took place in the TSE during the past decade.

**Fig 2:**
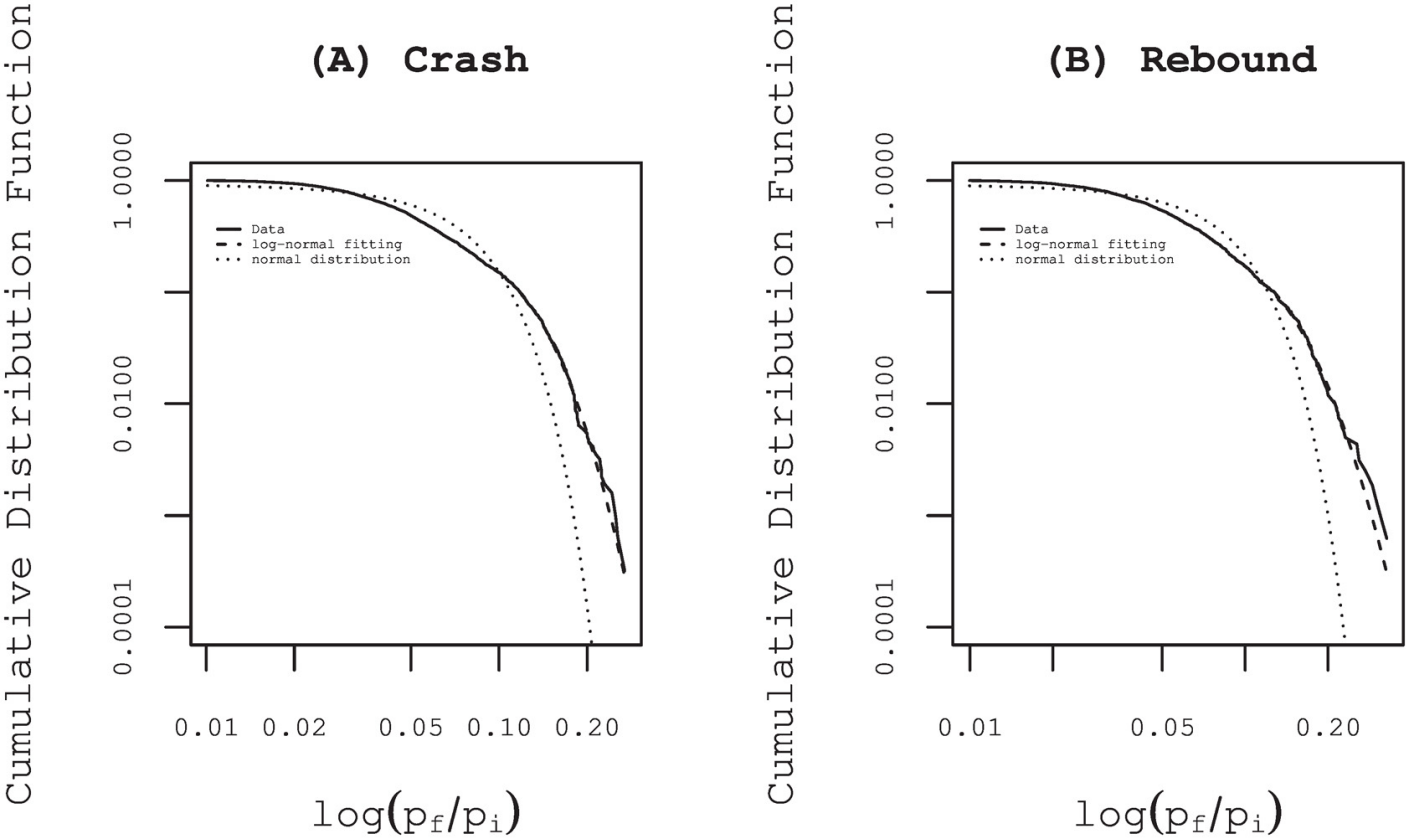
Cumulative distribution function of the log-returns during special quotes. Panels (A) and (B) show cumulative distribution functions (CDFs) of log-returns ln(*P*_*f*_/*P*_*i*_) during the total 3126 special quotes for crashes and during the total 1600 ones for rebounds respectively, where *P*_*f*_ and *P*_*i*_ express the final and the initial price of the special quote. Both cases of CDFs are well fitted by the same lognormal distributions function (dashed line), although the normal distribution function is not good for the statistical model (dotted line).

The log-returns during the special quote, which is proportional to the duration time of special quote, widely distribute beyond 20% (Fig 2).

All but the crash and rebound on 15 March 2011 took place immediately after the opening of the morning session, suggesting that many market participants have shared a vague expectation about the price movements of those days before the openings. The news coverage of the “Fukushima Daiichi nuclear disaster” was provided in the morning on 15 March 2011, then the second largest crash occurred immediately after opening of the afternoon session. In both cases, market participants did not know whether a crash or a rebound would occur nor how much the price change would be. Therefore, they were forced to place orders with no confidence. Therefore, the behavior depending on order placements of other market participants would emerge among them. Here we would like to analyze the interaction between market participants under such uncertain situations by measuring the order flow during the special quotes.

### Analysis

**Fig 3:**
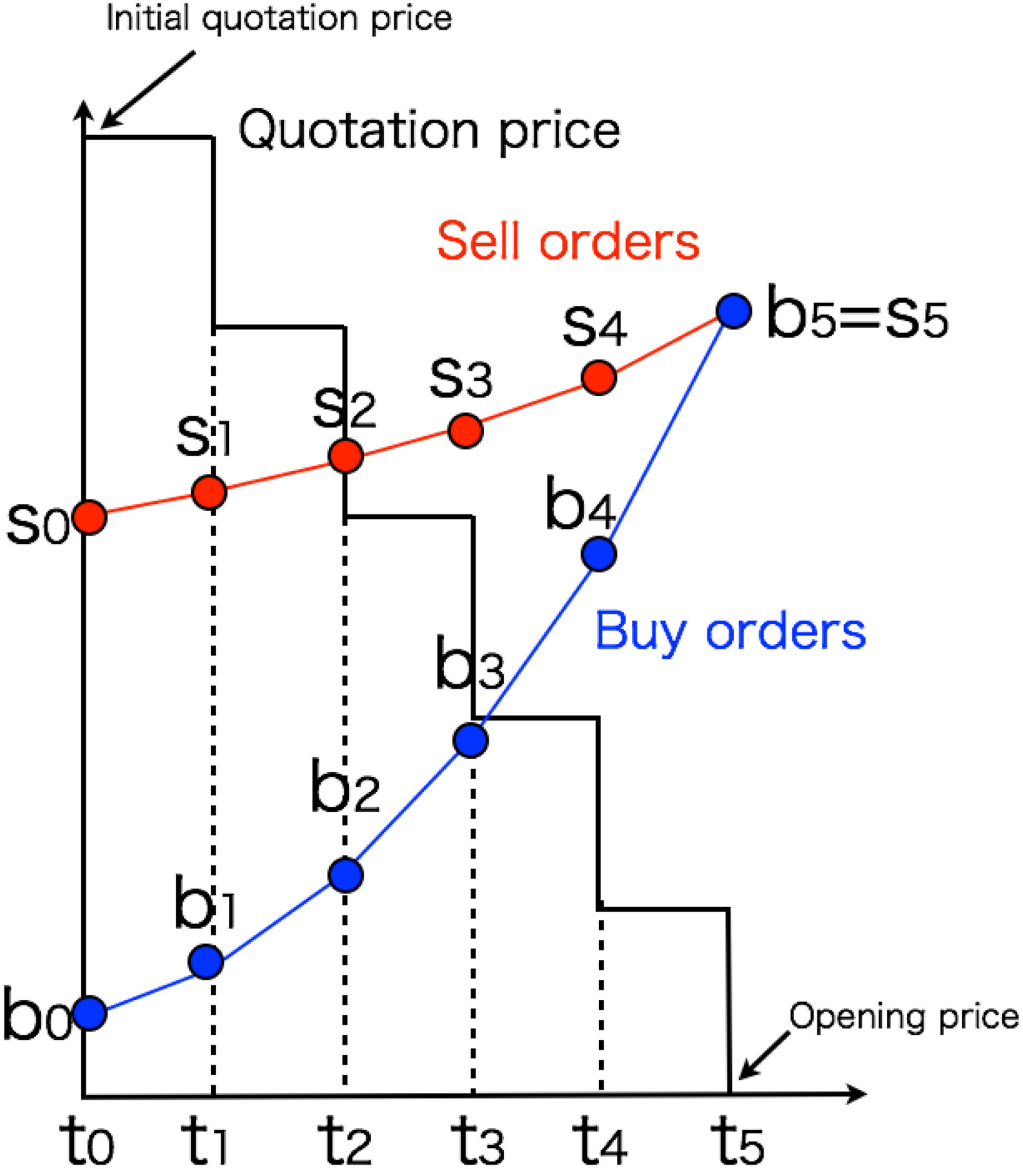
Schematic picture of the process of special quote in discrete time. An example is shown for the case of a price decline. Time *t*_0_ is the time when the special quote start and the time *t*_*i*_ (*i* = 1, 2, …, 5) expresses the *i*_*th*_ renewal of the quote price. The buy (sell) order *b*_*i*_ (*s*_*i*_) (*i* = 0, 2, …, 5) denotes the quantity of order placed by market participants who want to buy (sell) below (above) the price at the time *t*_*i*_ in the unit of block of shares. In the case of this picture, *b*_5_ (= *s*_5_) is the final equilibrium buy order at the end of the special quote.

Market participants can place their orders anytime they wish during the special quote, whereas the quote price is renewed at fixed time intervals. Let the time updating the quote price be denoted as *t*_*i*_ (*i* = 0, 1, 2, …, f), where *t* = *t*_0_ and *t*_*f*_ respectively denote the time of onset and termination time of the special quote. We analyze the quantities of buy and sell orders *b*_*i*_ and *s*_*i*_ of each stock at discrete time *t* = *t*_*i*_ for simplicity. Quantity *b*_*i*_ (*s*_*i*_) denotes the accumulated total orders of which the limit price less (greater) or equal to the quote price at the time *t*_*i*_, i.e., the buy (sell) orders *b*_*i*_ − *b*_*i*−1_ (*s*_*i*_ − *s*_*i*−1_) of the unrevealed orders *b*_*f*_ − *b*_*i*−1_ (*s*_*f*_ − *s*_*i*−1_) have been revealed during the time interval *t*_*i*_ − *t*_*i*−1_ in the unit of block of shares. The size of a block varies by issue. A schematic image of the process of special quote in discrete time is depicted in Fig 3.

As described in this paper, we would like to prove that each market participant is subject to influence from the decisions of other participants when placing orders under uncertain and emergent situations such as special quote during market crashes or rebounds. To this end, we demonstrate that the probability of placing balancing orders by a market participant with each passing time of the special quotes increases with the accumulated orders in the book until then.

The total order quantities placed during the time interval *t*_*i*_ − *t*_*i*−1_ would be limited by a ceiling determined by the potential orders which might not be recognized by each participant. Here we do not consider the remaining orders unrevealed until the termination of each special quote and define the limit by the quantity *b*_*f*_ − *b*_*i*−1_ (*s*_*f*_ − *s*_*i*−1_). We assume that each order is placed independently with equal probability. Therefore, the orders *b*_*i*_ − *b*_*i*−1_ (*s*_*i*_ − *s*_*i*−1_) (i = 1, 2, …, f-1) are assumed to follow a binomial distribution,

P(bi-bi-1|bf-bi-1,πi)=bf-bi-1bi-bi-1πibi-bi-1(1-πi)bf-bi(b↔s). (1)

Here, the logit function $\text{logit}\left(\pi_{i}\right)=\log\left(\pi_{i}/\left(1-\pi_{i}\right)\right)$ of the probability of success is assumed to be a linear function of the revealed ratio *r*_*i*_ = *b*_*i*−1_/*b*_*f*_ (*r*_*i*_ = *s*_*i*−1_/*s*_*f*_) until time *t* = *t*_*i*−1_, i.e.,

$$\pi_{i}=\frac{\exp(\beta_{0}+\beta_{1}*r_{i})}{1+\exp(\beta_{0}+\beta_{1}*r_{i})}.$$ (2)

The right-hand side of the equation is a logistic function which assure the condition 0 ≤ *π*_*i*_ ≤ 1 and also that the *π*_*i*_ increases monotonically with *r*_*i*_ if the parameter *β*_1_ is positive, which means that revealed balancing counter orders increase the probability of such coming orders. Parameters *β*_0_ and *β*_1_ will be estimated for each special quote for each stock using maximum likelihood method. This is a logistic regression model [32] in which the explanatory variable is the coming buy (sell) orders *b*_*i*_ − *b*_*i*−1_ (*s*_*i*_ − *s*_*i*−1_) of the unrevealed orders *b*_*f*_ − *b*_*i*−1_ (*s*_*f*_ − *s*_*i*−1_). We will show that, for almost all cases, parameter *β*_1_ is significantly positive and that the revealed buy (sell) orders at the time *t* = *t*_*i*_ are well described by the equation,

$$E\left[b_{i}\right]=\pi_{i}\left(b_{f}-b_{i-1}\right)+b_{i-1}\;\left(b↔s\right).$$ (3)

We adopt the finally revealed orders *b*_*f*_(*s*_*f*_) as the normalization factor for the explanatory variable *r*_*i*_. The choice of the normalization factor is irrelevant to the result. Even if we choose another explanatory variable ${\tilde{r}}_{i}=b_{i-1}/b_{0}$ $\left({\tilde{r}}_{i}=s_{i-1}/s_{0}\right)$ like $\text{logit}(\pi_{i})=\beta_{0}+{\tilde{\beta }}_{1}*{\tilde{r}}_{i}$, we can easily convert the result obtained using the relation of those estimated values of the coefficients ${\tilde{\beta }}_{1}=\beta_{1}*b_{0}/b_{f}$ $\left({\tilde{\beta }}_{1}=\beta_{1}*s_{0}/s_{f}\right)$. The choice of the explanatory variable *r*_*i*_ is convenient to show the results for all special quotes with different range of order *b*_*i*_ (*s*_*i*_) against the same range of 0 ≤ *r*_*i*_ ≤ 1.

## Results

**Fig 4:**
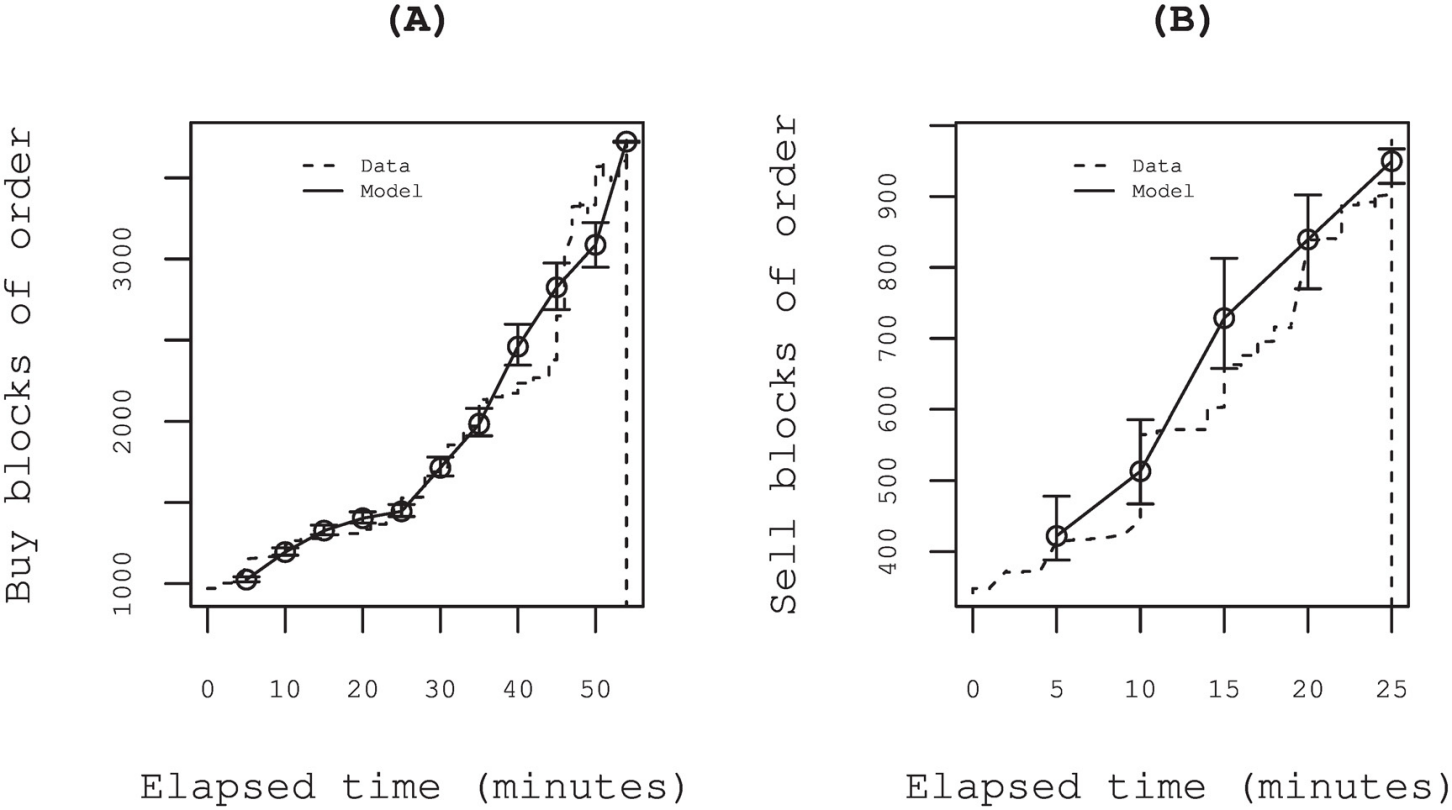
Examples of fitting by the logistic model. Evolution of revealed orders *E*[*b*_*i*_] and *E*[*s*_*i*_] predicted using Eq (3) are shown. The error bar denotes the standard error evaluated by the inverse of the observed information matrix [32]. (A) Example from the market crash on 16 Oct. 2008. The issue is Toyota Tsusho Corp. (Trading Co.): *β*_0_ = 6.5 and *β*_1_ = −10.1. (B) An example from the market rebound on 14 Oct. 2008. The issue is Oki Electric Ind. Co. Ltd. (Electric Machinery): *β*_0_ = 4.5 and *β*_1_ = −7.0.

We estimated parameters *β*_0_ and *β*_1_ of the logistic regression models (1) and (2) using maximum likelihood method. In Fig 4 we first show two examples of the parameter estimations for the same special quotes as in Fig 1. Panels (A) and (B) respectively show the evolution of revealed orders *E*[*b*_*i*_] and *E*[*s*_*i*_] predicted by Eq (3). The error bar denotes the standard error evaluated by the inverse of the observed information matrix [32]. The revealed orders up to discrete time *t* = *t*_*i*_ are well described within the standard deviations denoted by error bars.

We specifically examine special quotes for which the price was renewed more than four times in consideration of the statistical precision. Among all 3126 (1600) target special quotes, 1070 (661) such special quotes occurred during crashes (rebounds). The total 1069 (661) estimated parameters *β*_0_ are negative, which amount to 99.0% (100%) of the 1070 (661) special quotes. Furthermore, the total 1069 (660) estimated parameters *β*_1_ are positive, amounting to 99.9% (99.8%) of the 1070 (661) special quotes. Negative *β*_0_ means that the prior probability of placing counter orders for market participants is less than 50% when no counter orders are revealed. The average of the 1069 (661) estimated parameters *β*_0_ is -6.86 (-9.86). The average probability is 2.6% (1.1%). Positive *β*_1_ means that the posterior probability increases with the increase of the revealed orders.

$$\frac{d\pi_{i}}{dr_{i}}=\frac{\beta_{1}\exp\left(\beta_{0}+\beta_{1}*r_{i}\right)}{{\left(1+\exp\left(\beta_{0}+\beta_{1}*r_{i}\right)\right)}^{2}}$$ (4)

**Fig 5:**
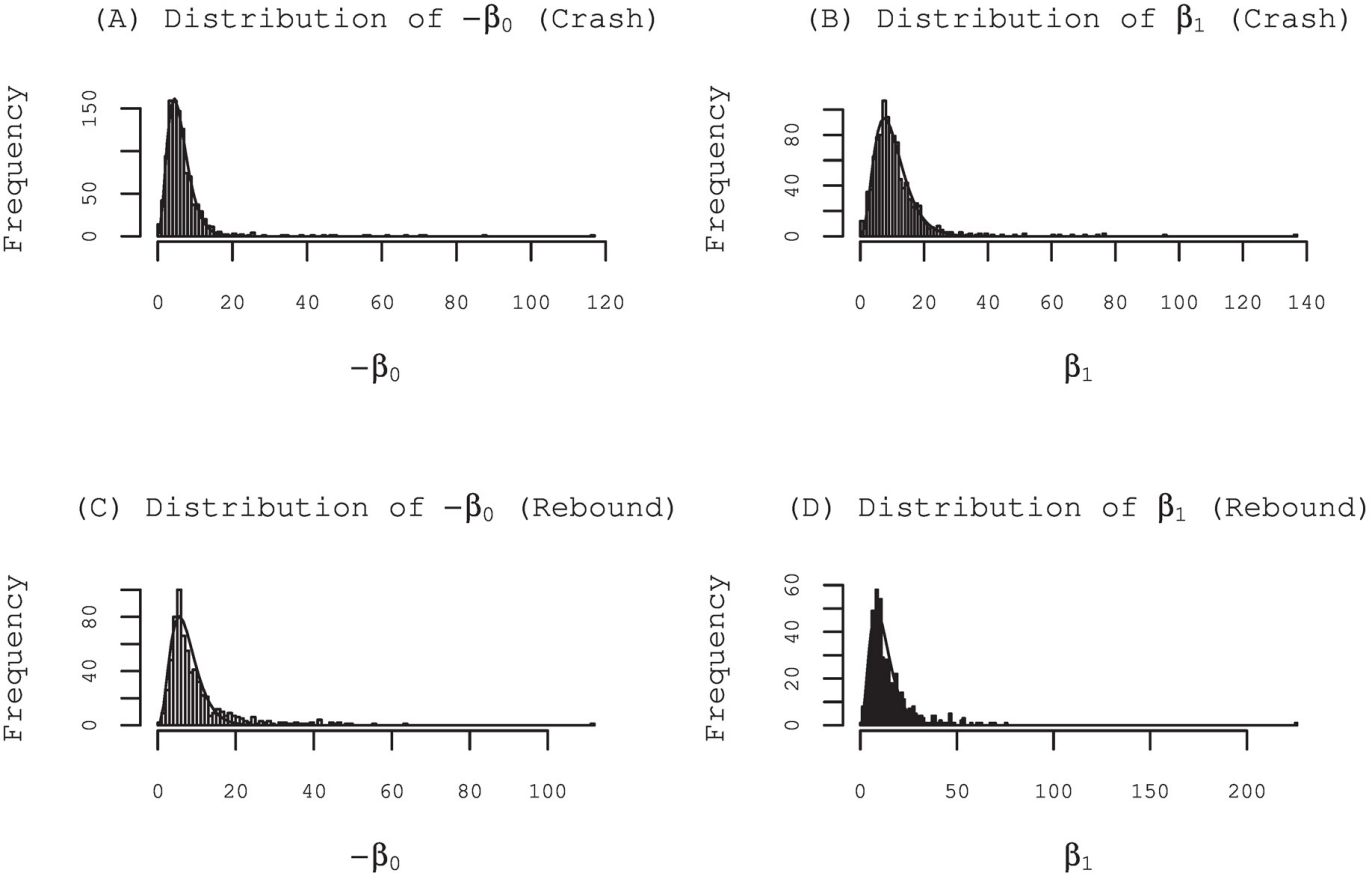
Distribution of the parameters estimated using maximum-likelihood method. For the special quotes in times of crashes, the estimated values of the parameter *β*_0_ are negative, with one exception. The parameters *β*_1_ are positive with one exception. Upper panels (A) and (B) show a distribution of the parameters *β*_0_ and *β*_1_ of the model for the 1069 special quotes under crashes excluding those exception. For the special quotes under rebounds, the estimated values of the parameter *β*_1_ are positive with the exception of only a single case. Lower panels (C) and (D) are those for the 660 special quotes under rebound excluding the exception. All the distribution functions are well fitted by the Gamma distribution *f*(*x*) = *x*^*k*−1^ exp(−*x*/*θ*)/Γ(*k*)/*θ*^*k*^ with different parameters *k* and *θ* (solid line): (A) *k* = 3.9, *θ* = 1.5; (B) *k* = 3.9, *θ* = 2.6; (C) *k* = 4.0, *θ* = 1.8; and (D) *k* = 3.6, *θ* = 3.2.

The average of the 1069 (661) estimated parameters *β*_1_ is 11.0 (14.3) and a block of counter orders by the other participant averagely pushes the probability up about 9.1% (4.1%) according to Eq (4) (*r*_*i*_ = 0). Fig 5 shows that those values seem to vary from issue to issue and depending on the economical situation when the crash or rebound takes place. Thsy are broadly distributed following a Gamma distribution. Tables of the average values of the parameters estimated using maximum-likelihood method are presented in Tables in S1 File. The common distinguishing feature of those special quotes is the collective behavior of market participants expressed as the positivity of the estimated parameters *β*_1_.

**Fig 6:**
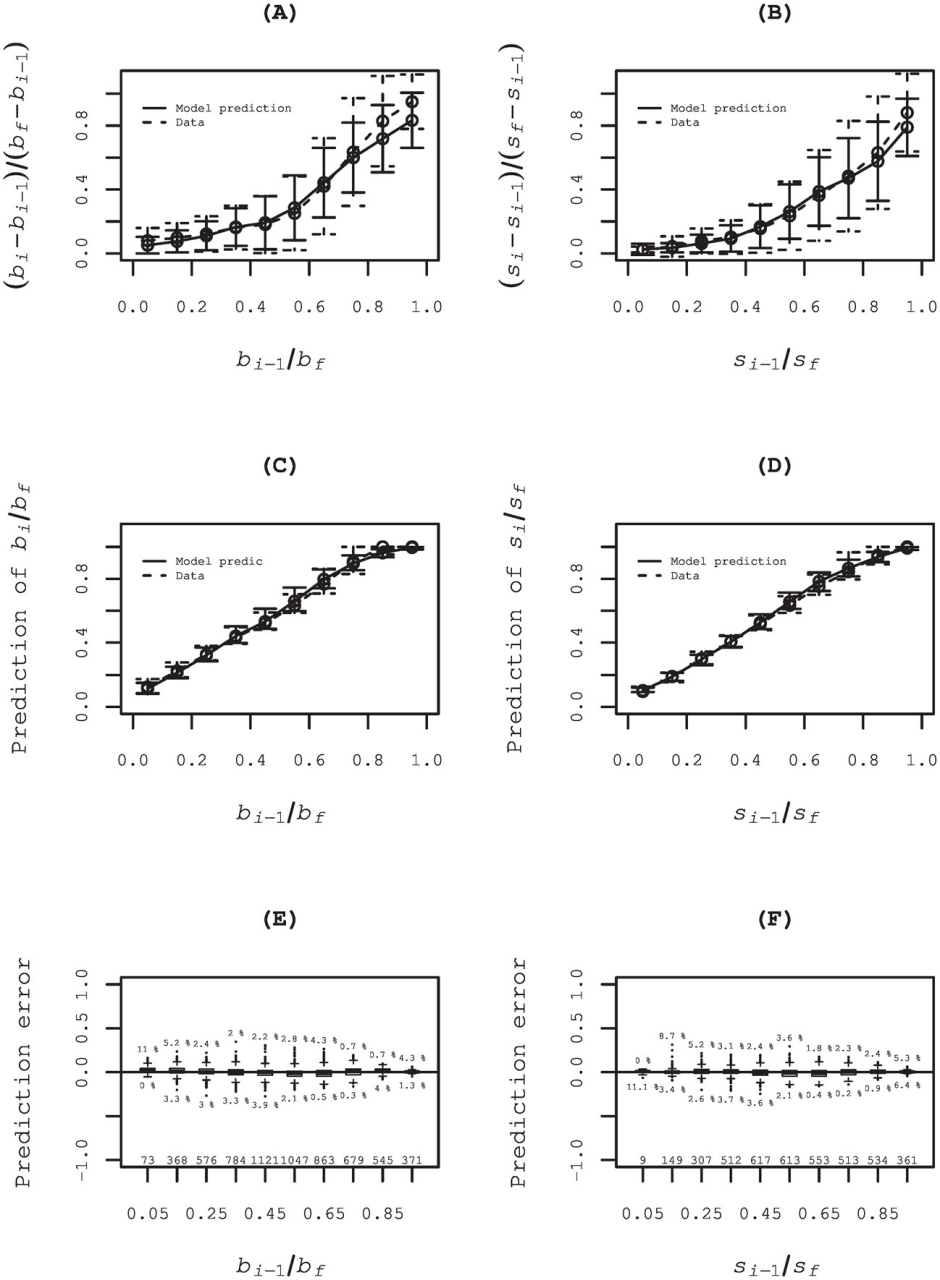
Model prediction of coming order by the model. In upper panels (A) and (B), we divide the range [0, 1] of the ratio *b*_*i*−1_/*b*_*f*_ (*s*_*i*−1_/*s*_*f*_) into 10 bins of equal length. Those panels show the average (circle) and the standard deviation (bar) of the ratio of the coming order to the unrevealed order, i.e., (*b*_*i*_ − *b*_*i*−1_)/(*b*_*f*_ − *b*_*i*−1_) in (A) and (*s*_*i*_ − *s*_*i*−1_)/(*s*_*f*_ − *s*_*i*−1_) in (B), in each bin. The solid and dashed line respectively show the result estimated using the model and the corresponding data. In panels (C)–(F), the range [0, 1] of the ratio *b*_*i*−1_/*b*_*f*_ (*s*_*i*−1_/*s*_*f*_) is divided into 10 bins of equal length. Middle panels (C) and (D) show the median (circle) and the IQR (bar) of the revealed order until the *i*-th step in each bin of the actual ratio, i.e., *b*_*i*_/*b*_*f*_ in (C) and *s*_*i*_/*s*_*f*_ in (D), and the prediction, i.e., *E*[*b*_*i*_]/*b*_*f*_ in (C) and *E*[*s*_*i*_/*s*_*f*_] in (D). Lower panels (E) and (F) show the box plot of the prediction error, i.e., *b*_*i*_/*b*_*f*_ − *E*[*b*_*i*_]/*b*_*f*_ in (E) and *s*_*i*_/*s*_*f*_ − *E*[*s*_*i*_]/*s*_*f*_ in (F), in each bin. The integers above the horizontal axes express the numbers of data in respective bins. The ratios of the outliers larger than *Q*_3/4_+1.5*IQR* and the outliers less than *Q*_1/4_ − 1.5*IQR* are attached respectively above and below the boxes.

Upper panels (A) and (B) in Fig 6 show the average (circle) and the standard deviation (bar) of the actual ratio of the coming order, i.e., (*b*_*i*_ − *b*_*i*−1_)/(*b*_*f*_ − *b*_*i*−1_) in (A) and (*s*_*i*_ − *s*_*i*−1_)/(*s*_*f*_ − *s*_*i*−1_)/ in (B), in the bins of the ratio *b*_*i*−1_/*b*_*f*_ (*s*_*i*−1_/*s*_*f*_). Middle panels (C) and (D) show the median (circle) and the IQR (bar) of the revealed order until the *i*-th step in the bins of the actual ratio, i.e., *b*_*i*_/*b*_*f*_ in (C) and *s*_*i*_/*s*_*f*_ in (D), and the prediction, i.e., *E*[*b*_*i*_]/*b*_*f*_ in (C) and *E*[*s*_*i*_]/*s*_*f*_ in (D). Lower panels (E) and (F) show the box plot of the prediction error, i.e., *b*_*i*_/*b*_*f*_ − *E*[*b*_*i*_]/*b*_*f*_ in (E) and *s*_*i*_/*s*_*f*_ − *E*[*s*_*i*_]/*s*_*f*_ in (F), in each bin. Results indicate fairly good precision of the model predictions. Actually, 72% (77%) of all prediction errors at all steps during buy (sell) special quotes are within 5% and the 92% (95%) is within 10%.

**Fig 7:**
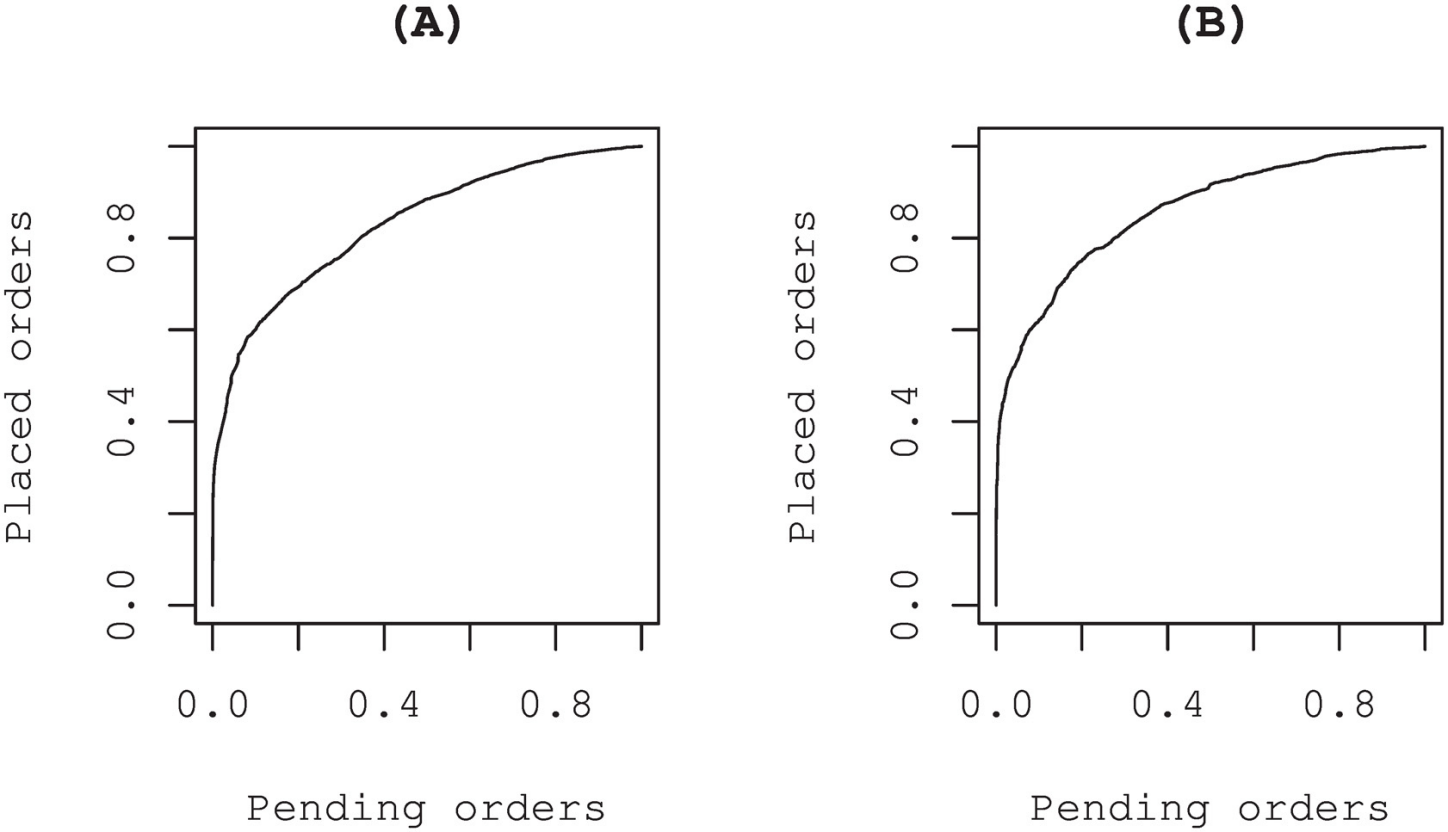
Goodness of fit of the model: ROC curve and the AUC. Panels (A) and (B) show the receiver operating characteristic (ROC) curves for buy and sell special quotes. We arranged the sets of placed buy (sell) orders *b*_*i*_ − *b*_*i*−1_ (*s*_*i*_ − *s*_*i*−1_) and pending orders *b*_*f*_ − *b*_*i*_ (*s*_*f*_ − *s*_*i*_) at each of all steps in all special quotes under study in ascending order of the estimated probability *π*_*i*_: *π*_(1)_ < *π*_(2)_ < … < *π*_(*n*)_ with the number of all steps denoted by n. Each point expresses the pair of quantities (∑_*ist*. *π*_*i*_ ≥ *π*_(*j*)__(*b*_*f*_ − *b*_*i*_)/∑_*i*_(*b*_*f*_ − *b*_*i*_), ∑_*ist*. *π*_*i*_ ≥ *π*_(*j*)__(*b*_*i*_ − *b*_*i*−1_)/∑_*i*_(*b*_*i*_ − *b*_*i*−1_)) (*b* ↔ *s*) (*j* = 1, 2, …, *n*). (A) AUC = 0.83. (B) AUC = 0.86.

Panels (A) and (B) in Fig 7 respectively show the receiver operating characteristic (ROC) curves for buy and sell special quotes. Each point on the curve expresses the pair of aggregate quantities (the proportion of placed orders, the proportion of pending orders) at each of all steps in all special quotes under study over a given threshold *π* = *π*_*i*_. The area under the curve (AUC), which is equivalent to Wilcoxon statistics, is commonly used as the goodness of fit measure of the model as *R*^2^ for linear regressions [33]. Those results also show that the revealed order until a step of the special quote is an appropriate variable for characterizing each step of the process.

## Discussion

We investigated the process of clearing extreme supply-and-demand order imbalances by the increase of counter orders. i.e., buy and sell orders for excess supply and demand respectively, during special quotes: a time of price adjustment. Counter orders have increased positively depending on the quantity of revealed counter orders, i.e., the accumulated orders in the order book up to that point. The statistics of the coming counter order are well described using a logistic regression model with the ratio of revealed order until then to finally revealed orders as the explanatory variable.

### Analysis using other models

For comparison, we also analyze the order flow by the Poisson regression model [32] in which the orders *b*_*i*_ − *b*_*i*−1_ (*s*_*i*_ − *s*_*i*−1_) (i = 1, 2, …, f-1) are not limited by a ceiling, i.e., unlimited potential orders, and follow a Poisson distribution, as

$$P\left(b_{i}-b_{i-1}|\mu_{i}\right)=\frac{\mu_{i}^{\left(b_{i}-b_{i-1}\right)}e^{-\mu_{i}}}{(b_{i}-b_{i-1})!}\;\left(b↔s\right).$$ (5)

Here, the logarithm of the parameter *μ*_*i*_ is assumed to be a linear function of the revealed ratio *r*_*i*_ = *b*_*i*−1_/*b*_*f*_ (*r*_*i*_ = *s*_*i*−1_/*s*_*f*_) until time *t* = *t*_*i*−1_, i.e.,

$$\mu_{i}=\exp\left({\tilde{\beta }}_{0}+{\tilde{\beta }}_{1}*r_{i}\right).$$ (6)

**Fig 8:**
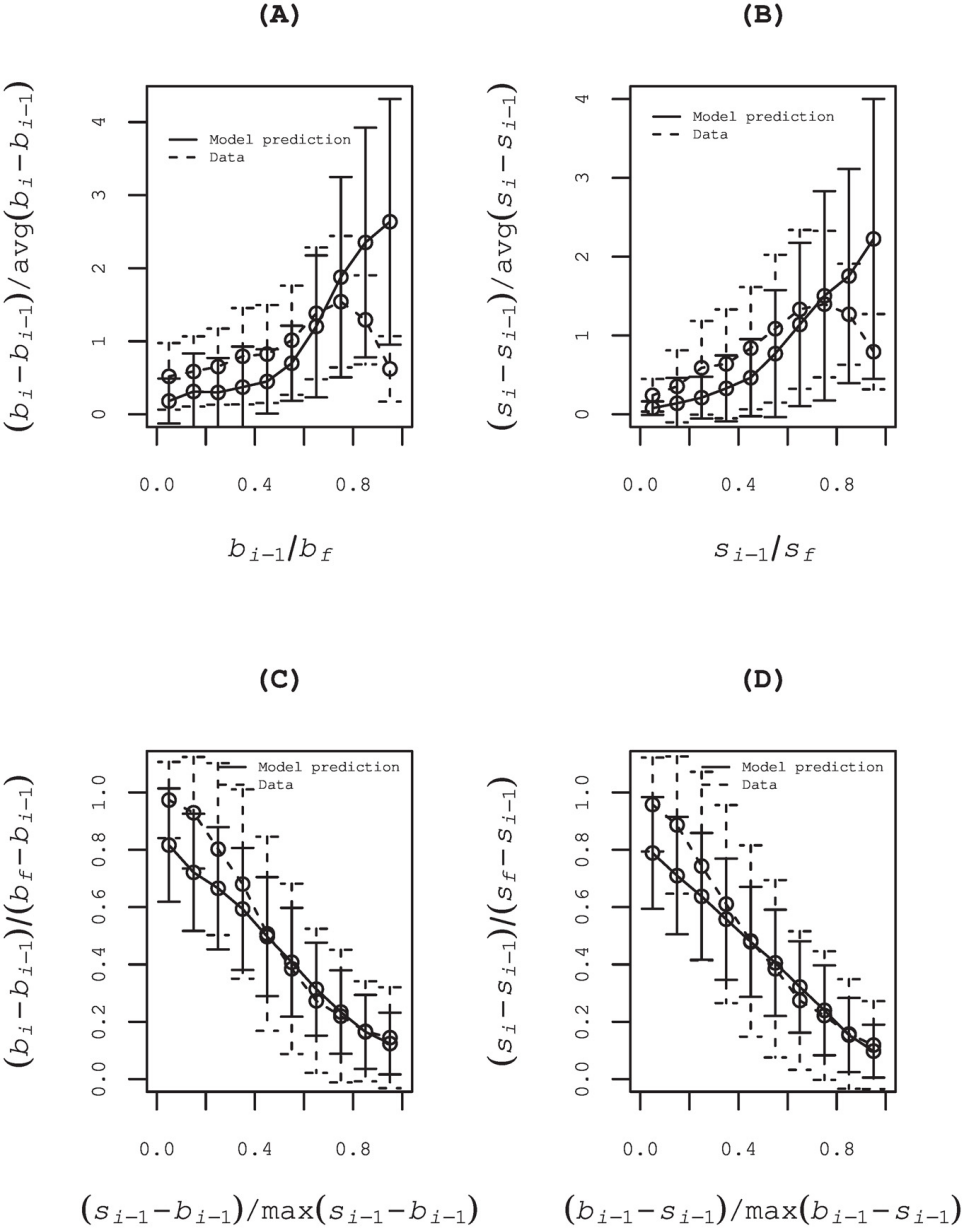
Results of data analysis using alternative models. In panels (A) and (B), the range [0, 1] of the ratio *b*_*i*−1_/*b*_*f*_ (*s*_*i*−1_/*s*_*f*_) is divided into 10 bins of equal length. Those panels show the average (circle) and the standard deviation (bar) of the coming order normalized by the average of coming orders at all the time of price update during each special quote, i.e., (*b*_*i*_ − *b*_*i*−1_)/*Avr*.(*b*_*i*_ − *b*_*i*−1_) in (A) and (*s*_*i*_ − *s*_*i*−1_)/*Avr*.(*s*_*i*_ − *s*_*i*−1_) in (B), in each bin. The solid and dashed line respectively show to the results estimated using the Poisson regression model and the corresponding actual data. In panels (C) and (D), the results of data analysis by the logistic regression model are shown with the supply-demand gap *s*_*i*−1_ − *bi*−1 (*b*_*i*−1_ − *si*−1) as the explanatory variable. Those gaps are normalized by the maximum gap during each special quote. In those panels, the range [0, 1] of the normalized gap (*s*_*i*−1_ − *b*_*i*−1_)/*Max*.(*s*_*i*−1_ − *b*_*i*−1_) ((*b*_*i*−1_ − *s*_*i*−1_)/Max.(*b*_*i*−1_ − *s*_*i*−1_)) is divided into 10 bins of equal length. Those panels show the average (circle) and the standard deviation (bar) of the ratio of the coming order to the unrevealed order, i.e., (*b*_*i*_ − *b*_*i*−1_)/(*b*_*f*_ − *b*_*i*−1_) in (C) and (*s*_*i*_ − *s*_*i*−1_)/(*s*_*f*_ − *s*_*i*−1_) in (D), in each bin. The solid and dashed line respectively show the result estimated using the model and the corresponding data.

The parameter *μ*_*i*_ increases monotonically with *r*_*i*_ if the parameter ${\tilde{\beta }}_{1}$ is positive in the same way as the logistic regression model, which means a collective behavior of market participants. The total 792 (501) estimated parameters ${\tilde{\beta }}_{1}$ are positive, which amount to 74% (76%) of all 1070 (661) special quotes for which the price was renewed more than four times. Results of data analysis using this model are shown in panels (A) and (B) of Fig 8. Those panels show that the model prediction of the coming order normalized by the average of coming orders at all the time of price update during each special quote deviate form data, especially for the range with high ratio *r*_*i*_. The amount of coming order tend to decrease over the peak around *r*_*i*_ ∼ 0.7, which means that they are limited by a ceiling of unrevealed order at the time.

We also have tried a supply-demand gap *s*_*i*−1_ − *b*_*i*−1_ (*b*_*i*−1_ − *s*_*i*−1_) as an alternative explanatory variable for the logistic regression model, which does not necessarily decrease monotonically but which eventually converges to zero. It is convenient to be normalized by the maximum gap during each special quote as ${\hat{r}}_{i}=\left(s_{i-1}-b_{i-1}\right)/Max.\left(s_{i-1}-b_{i-1}\right)$ (${\hat{r}}_{i}=\left(b_{i-1}-s_{i-1}\right)/Max.\left(b_{i-1}-s_{i-1}\right)$). The probability of success *π*_*i*_ is assumed to be expressed as the following.

$$\pi_{i}=\frac{\exp({\hat{\beta }}_{0}+{\hat{\beta }}_{1}*{\hat{r}}_{i})}{1+\exp({\hat{\beta }}_{0}+{\hat{\beta }}_{1}*{\hat{r}}_{i})}$$ (7)

Coefficient ${\hat{\beta }}_{1}$ is expected to be negative, which means market participants are likely to place orders when the gap becomes small. Actually, the total 1012 (632) estimated parameters ${\hat{\beta }}_{1}$ are negative, amounting to 95% (96%) of all 1070 (661) special quotes. That has also proved a collective behavior of market participants, i.e., the order supplying a demand creates more such orders. The results of data analyses using this model are shown in panels (C) and (D) of Fig 8. As shown in those panels, the model prediction of the coming order normalized by the unrevealed order at the time shows some deviation form data around the range with low ratio ${\hat{r}}_{i}$, i.e., near the final stage of special quote.

From the perspective of the fitness of the model to data, the logistic regression model with the revealed order as the explanatory variable shows the best performance among the three models investigated here. Actually, the average residual square errors (RSE’s) of the coming order normalized by the unrevealed order at the time are, respectively, 0.12 (0.14), 0.22 (0.21) and 18.5 (28.0) for the logistic model with the revealed order as the explanatory variable, for the logistic model with the supply-demand gap as the explanatory variable and the Poisson model with the revealed order as the explanatory variable. In this paper, our goal is to show collective behavior among market participants during special quotes. Therefore, we would not optimize a model describing the statistics of oder flows.

### Bayesian decision-making model

As described already in the Introduction, animals frequently make probability estimations related to optimal choices in uncertain situations. Animals use information obtained from the behaviors of other animals as well as their own information. Pérez-Escudero and de Polavieja build a Bayesian probability estimation model to analyze the experiment involving a group of fish [3–5]. Assuming two choices *X* (‘go to x’) and *Y* (‘go to y’), they derive the expression for the conditional probability *P*(*Y*|*C*, *B*) by which the choice *Y* is the optimal on the premise of private information *C* and the behavior of the other individuals *B* as shown below.

$$P\left(Y|C,B\right)=\frac{1}{1+a\prod_{k=1}^{L}s_{k}^{n_{k}}}$$ (8)

This equation deals with the case *L* different behavioral class ${\left\{b_{k}\right\}}_{k=1}^{L}$, e.g., ‘go to x’, ‘go to y’ and ‘remain undecided’, where *a* = *P*(*X*|*C*)/*P*(*Y*|*C*) is the likelihood-ratio for the two choices given only the personal information, where *n*_*k*_ is the number of individuals performing behavior *b*_*k*_ and

$$s_{k}=\frac{P(b_{k}|X,C)}{P(b_{k}|Y,C)},$$ (9)

where *P*(*b*_*k*_|*X*(*Y*), *C*) is the conditional probability that an individual behavior is *b*_*k*_ under the condition under which *X*(*Y*) is the optimal.

For dealing with our case, let choice *Y* be set as ‘put order’, *X* be set as ‘pending decision’ and *L* = 2, i.e., the behaviors *b*_1_ and *b*_2_ respectively corresponding to those choices *Y* and *X*.

$$P\left(Y|C,B\right)=\frac{1}{1+a^{\prime }s^{n_{1}}}$$ (10)

Therein, $a^{\prime }=as_{2}^{N}$,*s* = *s*_1_/*s*_2_ and the number of market participants is denoted as *N* = *n*_1_ + *n*_2_. If we assume that an individual chooses each option with probability *π*_*i*_ at each time *t* = *t*_*i*_ equal to the estimated probability *P*(*Y*|*C*, *B*), then Eq (2) can be interpreted as Eq (10) interpreting *a*′ = exp(−*β*_0_), *s* = exp(−*β*_1_/*b*_*f*_) and *n*1 = *b*_*i*_). Therefore, the positivity of parameter *β*_1_ means that *s* < 1 and the balancing orders by other participants raise the estimated probability that the choice *Y* (‘put order at the time *t* = *t*_*i*_’) is optimal.

### Future studies

**Fig 9:**
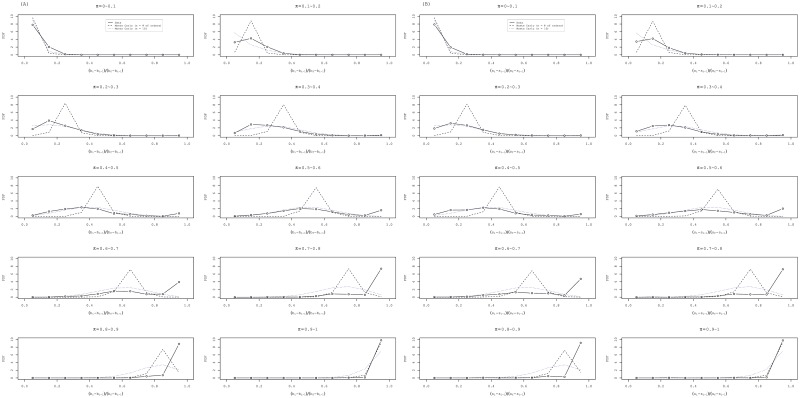
Probability distribution functions of the coming orders during the special quote. In groups (A) and (B) of panels, we divide the range [0, 1] of the estimated probability of success *π*_*i*_ into 10 bins of equal length. In each of panels, the probability distributions of coming buy (sell) order *b*_*i*_ − *b*_*i*−1_ (*s*_*i*_ − *s*_*i*−1_) are shown (rigid line), which is expected to be the binomial distribution with the average *π*_*i*_(*b*_*f*_ − *b*_*i*−1_) (*π*_*i*_(*s*_*f*_ − *s*_*i*−1_)). Random numbers are generated according to the binomial distributions with *p* = *π*_*i*_ as the probability of success and with the unrevealed orders *n* = *b*_*f*_ − *b*_*i*−1_ (*s*_*f*_ − *s*_*i*−1_) until the (*i*-1) step of special quote and the fixed number *n* = 10 as the number of trials. In each Monte Carlo simulation, 100 random numbers are generated. For comparison, the histograms of two series of random numbers, i.e., the case with *n* = *b*_*f*_ − *b*_*i*−1_ (*s*_*f*_ − *s*_*i*−1_) (dashed line) and with *n* = 10 (dotted line), are also shown in each panel. (A) Buy special quotes in crashes. (B) Sell special quotes in rebounds.

Comparison of the result of the analysis by the logistic regression model with the Bayesian decision-making model revealed that market participants use the information of the order placement of other participants to estimate the probability that revealing their order place at the time is the optimal choice. However, our data have no identifier for each market participant. Therefore, the unit in the application of the model must be a block of shares, although it is an individual fish in reports of the literature [3]. In Fig 9, we show the probability distributions of coming buy (sell) orders *b*_*i*_ − *b*_*i*−1_ (*s*_*i*_ − *s*_*i*−1_) in the bins of the estimated probability of success *π*_*i*_ (rigid line), which is expected to be the binomial distribution with the average *π*_*i*_(*b*_*f*_ − *b*_*i*−1_) (*π*_*i*_(*s*_*f*_ − *s*_*i*−1_)) if each block of order has been placed independently. For comparison, we conduct Monte Carlo simulations of two types. In one type of simulation, random numbers are generated according to binomial distributions with *π*_*i*_ and the unrevealed order *b*_*f*_ − *b*_*i*−1_ (*s*_*f*_ − *s*_*i*−1_) until the (*i*-1) step of special quote as the probability of success *p* and the number of trials *n* respectively (dashed line). In the other type of simulation, we use *p* = *π*_*i*_ and the fixed number of trials *n* = 10 (dotted line). It is apparent that the simulations with unrevealed orders *n* = *b*_*f*_ − *b*_*i*−1_ (*s*_*f*_ − *s*_*i*−1_) as the number of trials, which are typically several hundred, do not fit the data, although simulations with the fixed number *n* = 10, which is probably a usual round number of participants, do rather well.

**Fig 10:**
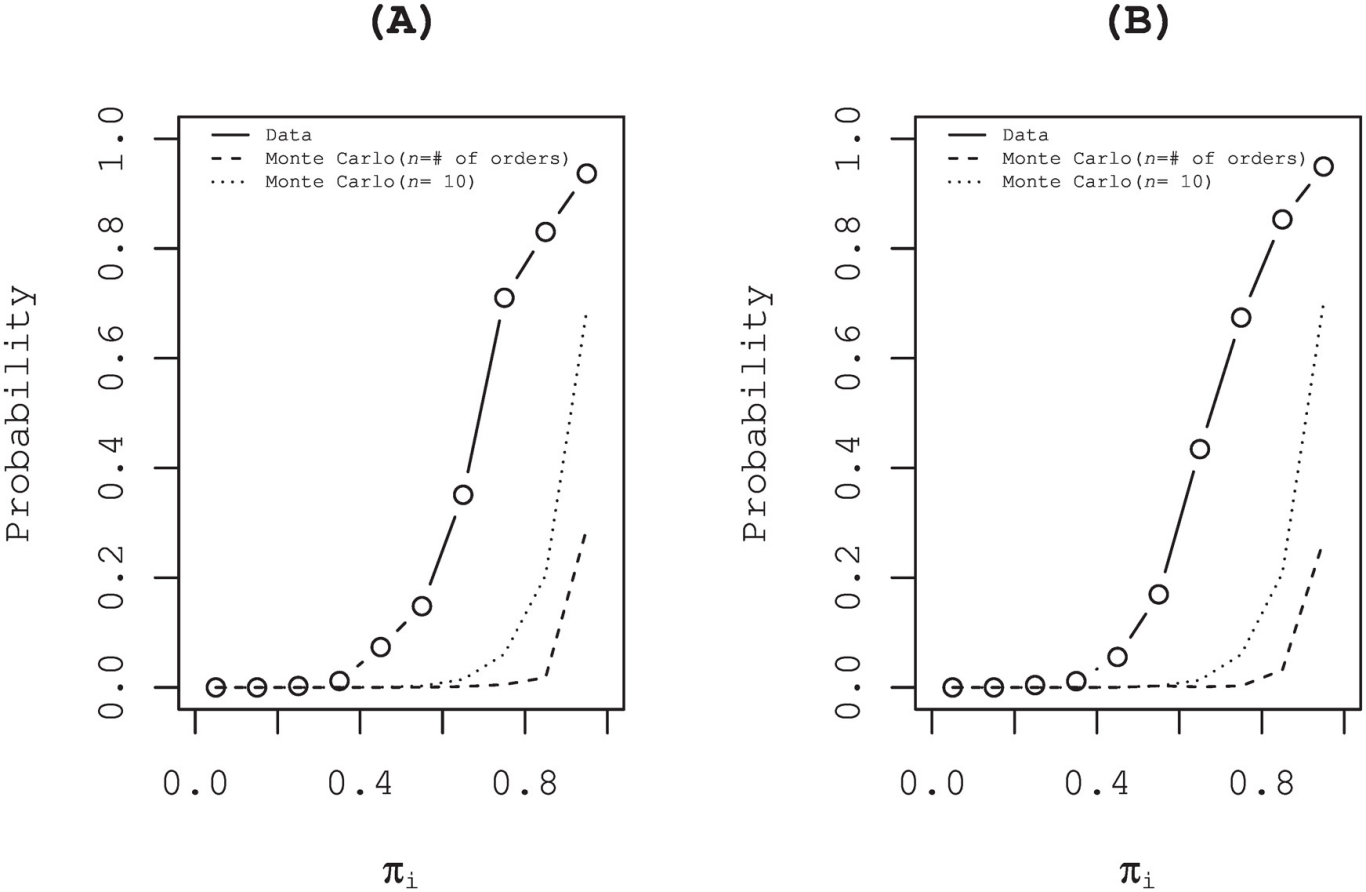
Information cascades during the special quote. The probability that all the unrevealed order until the (*i*-1)-th step of special quote are coming together at the *i*-th step are shown (rigid line). For comparison, results of Monte Carlo simulations explained in the legend of Fig 9 are also presented in the same panel. (A) Buy special quotes in crashes. (B) Sell special quotes in rebounds.

For the interpretation of results obtained using Bayesian estimation, we assume that an individual chooses each option with probability equal to the estimated probability [3]. Such an irrational manner of decision making is called probability matching. All rational individuals will choose the option when the estimated probability exceeds 50% without regarding one’s own information. That is, an information cascade will occur as explained in the Introduction. In both panels (A) and (B) of Fig 9, a local maximum of the curve of actual data appeared at the bin [0, 9, 1.0] in the panels where estimated probability *π* exceeding 0.5 might be a signal of an information cascade. The probability of all the unrevealed order until a time of a special quote coming together is presented in Fig 10 (rigid line). Implementing this behavior of market participants to the model is left as a task for future study.

## Supporting Information

S1 Table List of Nikkei 225 Index constituents in September 2015. (PDF)

S1 File Tables of average values of parameters estimated using maximum-likelihood method. (PDF)
