# Supplementary material for: Collective Behavior of Market Participants during Abrupt Stock Price Changes
Source: PLoS One. 2016 Aug 11;11(8):e0160152. doi: 10.1371/journal.pone.0160152 (PMC4981415; doi:10.1371/journal.pone.0160152)
Supplement: S1 Table — (PDF) [file pone.0160152.s001.pdf]

Table. Constituent issues of the Nikkei 225 Index at the moment of September 2015.

Code	Company	Category
4151	Kyowa Hakko Kirin Co. Ltd.	Pharmaceuticals
4502	Takeda Pharmaceutical Co. Ltd.	Pharmaceuticals
4503	Astellas Pharma Inc.	Pharmaceuticals
4506	Sumitomo Dainippon Pharma Co. Ltd.	Pharmaceuticals
4507	Shionogi & Co. Ltd.	Pharmaceuticals
4519	Chugai Pharmaceutical Co. Ltd.	Pharmaceuticals
4523	Eisai Co. Ltd.	Pharmaceuticals
4568	Daiichi Sankyo Co. Ltd.	Pharmaceuticals
6479	Minebea Co. Ltd.	Electric Machinery
6501	Hitachi Ltd.	Electric Machinery
6502	Toshiba Corp.	Electric Machinery
6503	Mitsubishi Electric Corp.	Electric Machinery
6504	Fuji Electric Co. Ltd.	Electric Machinery
6506	Yaskawa Electric Corp.	Electric Machinery
6508	Meidensha Corp.	Electric Machinery
6674	GS Yuasa Corp.	Electric Machinery
6701	NEC Corp.	Electric Machinery
6702	Fujitsu Ltd.	Electric Machinery
6703	Oki Electric Ind. Co. Ltd.	Electric Machinery
6752	Panasonic Corp.	Electric Machinery
6753	Sharp Corp.	Electric Machinery
6758	Sony Corp.	Electric Machinery
6762	TDK Corp.	Electric Machinery
6767	Mitsumi Electric Co. Ltd.	Electric Machinery
6770	Alps Electric Co. Ltd.	Electric Machinery
6773	Pioneer Corp.	Electric Machinery
6841	Yokogawa Electric Corp.	Electric Machinery
6857	Advantest Corp.	Electric Machinery
6902	Denso Corp.	Electric Machinery
6952	Casio Computer Co. Ltd.	Electric Machinery
6954	Fanuc Corp.	Electric Machinery
6971	Kyocera Corp.	Electric Machinery
6976	Taiyo Yuden Co. Ltd.	Electric Machinery

**7735**	**Screen Holdings Co. Ltd.**	**Electric Machinery**
**7751**	**Canon Inc.**	**Electric Machinery**
**7752**	**Ricoh Co. Ltd.**	**Electric Machinery**
**8035**	**Tokyo Electron Ltd.**	**Electric Machinery**
**7201**	**Nissan Motor Co. Ltd.**	**Automobiles & Auto parts**
**7202**	**Isuzu Motors Ltd.**	**Automobiles & Auto parts**
**7203**	**Toyota Motor Corp.**	**Automobiles & Auto parts**
**7205**	**Hino Motors Ltd.**	**Automobiles & Auto parts**
**7211**	**Mitsubishi Motors Corp.**	**Automobiles & Auto parts**
**7261**	**Mazda Motor Corp.**	**Automobiles & Auto parts**
**7267**	**Honda Motor Co. Ltd.**	**Automobiles & Auto parts**
**7269**	**Suzuki Motor Corp.**	**Automobiles & Auto parts**
**7270**	**Fuji Heavy Industries Ltd.**	**Automobiles & Auto parts**
**4543**	**Terumo Corp.**	**Precision Instruments**
**4902**	**Konica Minolta Inc.**	**Precision Instruments**
**7731**	**Nikon Corp.**	**Precision Instruments**
**7733**	**Olympus Corp.**	**Precision Instruments**
**7762**	**Citizen Holdings Co. Ltd.**	**Precision Instruments**
**9412**	**Sky Perfect JSAT Holdings Inc.**	**Communications**
**9432**	**Nippon Telegraph & Telephone Corp.**	**Communications**
**9433**	**KDDI Corp.**	**Communications**
**9437**	**NTT Docomo, Inc.**	**Communications**
**9613**	**NTT Data Corp.**	**Communications**
**9984**	**Softbank Group Corp.**	**Communications**
**8303**	**Shinsei Bank Ltd.**	**Banking**
**8304**	**Aozora Bank Ltd.**	**Banking**
**8306**	**Mitsubishi UFJ Financial Group Inc.**	**Banking**
**8308**	**Resona Holdings Inc.**	**Banking**
**8309**	**Sumitomo Mitsui Trust Holdings Inc.**	**Banking**
**8316**	**Sumitomo Mitsui Financial Group Inc.**	**Banking**
**8331**	**The Chiba Bank Ltd.**	**Banking**
**8332**	**The Bank Of Yokohama Ltd.**	**Banking**
**8354**	**Fukuoka Financial Group Inc.**	**Banking**
**8355**	**The Shizuoka Bank Ltd.**	**Banking**
**8411**	**Mizuho Financial Group Inc.**	**Banking**
**8253**	**Credit Saison Co. Ltd.**	**Other Financial Services**
**8601**	**Daiwa Securities Group Inc.**	**Securities**
**8604**	**Nomura Holdings Inc.**	**Securities**
**8628**	**Matsui Securities Co. Ltd.**	**Securities**
**8630**	**Sompo Japan Nipponkoa Holdings Inc.**	**Insurance**
**8725**	**MS&AD Insurance Group Holdings Inc.**	**Insurance**
**8729**	**Sony Financial Holdings Inc.**	**Insurance**
**8750**	**The Dai-Ichi Life Insurance Co. Ltd.**	**Insurance**

**8766**	**Tokio Marine Holdings Inc.**	**Insurance**
**8795**	**T&D Holdings Inc.**	**Insurance**
**1332**	**Nippon Suisan Kaisha Ltd.**	**Fishery**
**1333**	**Maruha Nichiro Corp.**	**Fishery**
**2002**	**Nisshin Seifun Group Inc.**	**Foods**
**2269**	**Meiji Holdings Co. Ltd.**	**Foods**
**2282**	**NH Foods Ltd.**	**Foods**
**2501**	**Sapporo Holdings Ltd.**	**Foods**
**2502**	**Asahi Group Holdings Ltd.**	**Foods**
**2503**	**Kirin Holdings Co. Ltd.**	**Foods**
**2531**	**Takara Holdings Inc.**	**Foods**
**2801**	**Kikkoman Corp.**	**Foods**
**2802**	**Ajinomoto Co. Inc.**	**Foods**
**2871**	**Nichirei Corp.**	**Foods**
**2914**	**Japan Tobacco Inc.**	**Foods**
**3086**	**J. Front Retailing Co. Ltd.**	**Retail**
**3099**	**Isetan Mitsukoshi Holdings Ltd.**	**Retail**
**3382**	**Seven & I Holdings Co. Ltd.**	**Retail**
**8233**	**Takashimaya Co. Ltd.**	**Retail**
**8252**	**Marui Group Co. Ltd.**	**Retail**
**8267**	**AEON Co. Ltd.**	**Retail**
**8270**	**UNY Group Holdings Co. Ltd.**	**Retail**
**9983**	**Fast Retailing Co. Ltd.**	**Retail**
**4324**	**Dentsu Inc.**	**Services**
**4689**	**Yahoo Japan Corp.**	**Services**
**4704**	**Trend Micro Inc.**	**Services**
**9602**	**Toho Co. Ltd**	**Services**
**9681**	**Tokyo Dome Corp.**	**Services**
**9735**	**Secom Co. Ltd.**	**Services**
**9766**	**Konami Corp.**	**Services**
**1605**	**Inpex Corp.**	**Mining**
**3101**	**Toyobo Co. Ltd.**	**Textiles & Apparel**
**3103**	**Unitika Ltd.**	**Textiles & Apparel**
**3105**	**Nisshinbo Holdings Inc.**	**Textiles & Apparel**
**3401**	**Teijin Ltd.**	**Textiles & Apparel**
**3402**	**Toray Industries, Inc.**	**Textiles & Apparel**
**3861**	**Oji Holdings Corp.**	**Pulp & Paper**
**3863**	**Nippon Paper Industries Co. Ltd.**	**Pulp & Paper**
**3865**	**Hokuetsu Kishu Paper Co. Ltd.**	**Pulp & Paper**
**3405**	**Kuraray Co. Ltd.**	**Chemicals**
**3407**	**Asahi Kasei Corp.**	**Chemicals**
**4004**	**Showa Denko K.K.**	**Chemicals**
**4005**	**Sumitomo Chemical Co. Ltd.**	**Chemicals**

**4021**	**Nissan Chemical Ind. Ltd.**	**Chemicals**
**4041**	**Nippon Soda Co. Ltd.**	**Chemicals**
**4042**	**Tosoh Corp.**	**Chemicals**
**4043**	**Tokuyama Corp.**	**Chemicals**
**4061**	**Denki Kagaku Kogyo K.K.**	**Chemicals**
**4063**	**Shin-Etsu Chemical Co. Ltd.**	**Chemicals**
**4183**	**Mitsui Chemicals Inc.**	**Chemicals**
**4188**	**Mitsubishi Chemical Holdings Corp.**	**Chemicals**
**4208**	**Ube Industries Ltd.**	**Chemicals**
**4272**	**Nippon Kayaku Co. Ltd.**	**Chemicals**
**4452**	**Kao Corp.**	**Chemicals**
**4901**	**Fujifilm Holdings Corp.**	**Chemicals**
**4911**	**Shiseido Co. Ltd.**	**Chemicals**
**6988**	**Nitto Denko Corp.**	**Chemicals**
**5002**	**Showa Shell Sekiyu K.K.**	**Petroleum**
**5020**	**JX Holdings Inc.**	**Petroleum**
**5101**	**The Yokohama Rubber Co. Ltd.**	**Rubber**
**5108**	**Bridgestone Corp.**	**Rubber**
**3110**	**Nitto Boseki Co. Ltd.**	**Glass & Ceramics**
**5201**	**Asahi Glass Co. Ltd.**	**Glass & Ceramics**
**5202**	**Nippon Sheet Glass Co. Ltd.**	**Glass & Ceramics**
**5214**	**Nippon Electric Glass Co. Ltd.**	**Glass & Ceramics**
**5232**	**Sumitomo Osaka Cement Co. Ltd.**	**Glass & Ceramics**
**5233**	**Taiheiyo Cement Corp.**	**Glass & Ceramics**
**5301**	**Tokai Carbon Co. Ltd.**	**Glass & Ceramics**
**5332**	**Toto Ltd.**	**Glass & Ceramics**
**5333**	**NGK Insulators Ltd.**	**Glass & Ceramics**
**5401**	**Nippon Steel & Sumitomo Metal Corp.**	**Steel**
**5406**	**Kobe Steel Ltd.**	**Steel**
**5411**	**JFE Holdings Inc.**	**Steel**
**5413**	**Nisshin Steel Co. Ltd.**	**Steel**
**5541**	**Pacific Metals Co. Ltd.**	**Steel**
**3436**	**Sumco Corp.**	**Nonferrous Metals**
**5703**	**Nippon Light Metal Holdings Co. Ltd.**	**Nonferrous Metals**
**5706**	**Mitsui Mining & Smelting Co.**	**Nonferrous Metals**
**5707**	**Toho Zinc Co. Ltd.**	**Nonferrous Metals**
**5711**	**Mitsubishi Materials Corp.**	**Nonferrous Metals**
**5713**	**Sumitomo Metal Mining Co. Ltd.**	**Nonferrous Metals**
**5714**	**Dowa Holdings Co. Ltd.**	**Nonferrous Metals**
**5715**	**Furukawa Co. Ltd.**	**Nonferrous Metals**
**5801**	**Furukawa Electric Co. Ltd.**	**Nonferrous Metals**
**5802**	**Sumitomo Electric Ind., Ltd.**	**Nonferrous Metals**
**5803**	**Fujikura Ltd.**	**Nonferrous Metals**

**5901**	**Toyo Seikan Group Holdings Ltd.**	**Nonferrous Metals**
**2768**	**Sojitz Corp.**	**Trading Companies**
**8001**	**Itochu Corp.**	**Trading Companies**
**8002**	**Marubeni Corp.**	**Trading Companies**
**8015**	**Toyota Tsusho Corp.**	**Trading Companies**
**8031**	**Mitsui & Co. Ltd.**	**Trading Companies**
**8053**	**Sumitomo Corp.**	**Trading Companies**
**8058**	**Mitsubishi Corp.**	**Trading Companies**
**1721**	**Comsys Holdings Corp.**	**Construction**
**1801**	**Taisei Corp.**	**Construction**
**1802**	**Obayashi Corp.**	**Construction**
**1803**	**Shimizu Corp.**	**Construction**
**1812**	**Kajima Corp.**	**Construction**
**1925**	**Daiwa House Ind. Co. Ltd.**	**Construction**
**1928**	**Sekisui House Ltd.**	**Construction**
**1963**	**JGC Corp.**	**Construction**
**5631**	**The Japan Steel Works Ltd.**	**Machinery**
**6103**	**Okuma Corp.**	**Machinery**
**6113**	**Amada Holdings Co. Ltd.**	**Machinery**
**6301**	**Komatsu Ltd.**	**Machinery**
**6302**	**Sumitomo Heavy Ind. Ltd.**	**Machinery**
**6305**	**Hitachi Const. Mach. Co. Ltd.**	**Machinery**
**6326**	**Kubota Corp.**	**Machinery**
**6361**	**Ebara Corp.**	**Machinery**
**6366**	**Chiyoda Corp.**	**Machinery**
**6367**	**Daikin Industries Ltd.**	**Machinery**
**6471**	**NSK Ltd.**	**Machinery**
**6472**	**NTN Corp.**	**Machinery**
**6473**	**JTEKT Corp.**	**Machinery**
**7004**	**Hitachi Zosen Corp.**	**Machinery**
**7011**	**Mitsubishi Heavy Ind. Ltd.**	**Machinery**
**7013**	**IHI Corp.**	**Machinery**
**7003**	**Mitsui Eng. & Shipbuild. Co. Ltd.**	**Shipbuilding**
**7012**	**Kawasaki Heavy Ind. Ltd.**	**Shipbuilding**
**7911**	**Toppan Printing Co. Ltd.**	**Other Manufacturing**
**7912**	**Dai Nippon Printing Co. Ltd.**	**Other Manufacturing**
**7951**	**Yamaha Corp.**	**Other Manufacturing**
**3289**	**Tokyu Fudosan Holdings Corp.**	**Real Estate**
**8801**	**Mitsui Fudosan Co. Ltd.**	**Real Estate**
**8802**	**Mitsubishi Estate Co. Ltd.**	**Real Estate**
**8803**	**Heiwa Real Estate Co. Ltd.**	**Real Estate**
**8804**	**Tokyo Tatemono Co. Ltd.**	**Real Estate**
**8830**	**Sumitomo Realty & Development Co. Ltd.**	**Real Estate**

**9001**	**Tobu Railway Co. Ltd.**	**Railway & Bus**
**9005**	**Tokyu Corp.**	**Railway & Bus**
**9007**	**Odakyu Electric Railway Co. Ltd.**	**Railway & Bus**
**9008**	**Keio Corp.**	**Railway & Bus**
**9009**	**Keisei Electric Railway Co. Ltd.**	**Railway & Bus**
**9020**	**Eastern Japan Railway Co.**	**Railway & Bus**
**9021**	**Western Japan Railway Co.**	**Railway & Bus**
**9022**	**Central Japan Railway Co. Ltd.**	**Railway & Bus**
**9062**	**Nippon Express Co. Ltd.**	**Land Transport**
**9064**	**Yamato Holdings Co. Ltd.**	**Land Transport**
**9101**	**Nippon Yusen K.K.**	**Marine Transport**
**9104**	**Mitsui O.S.K. Lines Ltd.**	**Marine Transport**
**9107**	**Kawasaki Kisen Kaisha Ltd.**	**Marine Transport**
**9202**	**ANA Holdings Inc.**	**Air Transport**
**9301**	**Mitsubishi Logistics Corp.**	**Warehousing**
**9501**	**Tokyo Electric Power Co. Inc.**	**Electric Power**
**9502**	**Chubu Electric Power Co. Inc.**	**Electric Power**
**9503**	**The Kansai Electric Power Co. Inc.**	**Electric Power**
**9531**	**Tokyo Gas Co. Ltd.**	**Gas**
**9532**	**Osaka Gas Co. Ltd.**	**Gas**
