# Supplementary material for: Collective Behavior of Market Participants during Abrupt Stock Price Changes
Source: PLoS One. 2016 Aug 11;11(8):e0160152. doi: 10.1371/journal.pone.0160152 (PMC4981415; doi:10.1371/journal.pone.0160152)
Supplement: S1 File — (PDF) [file pone.0160152.s002.pdf]

Tables of average values of the parameters estimated using maximum-likelihood method

Table 1: **Average values of parameters estimated using maximum-likelihood method for special quotes in crashes.**

	Date	β_0	β_1	Std. error of β_0	Std. error of β_1	*AIC*	*R2 MC*
1	10/16/2008	-6.28	10.96	0.28	0.56	531.36	0.74
2	3/15/2011	-2.67	4.46	0.17	0.33	3666.90	0.34
3	10/10/2008	-8.39	11.82	0.22	0.35	1961.57	0.68
4	10/24/2008	-5.95	9.65	0.42	0.72	324.55	0.65
5	10/8/2008	-7.74	12.60	0.62	0.99	331.75	0.68
6	5/23/2013	-	-	-	-	-	-
7	11/20/2008	-9.13	15.49	0.89	1.47	994.67	0.67
8	10/22/2008	-5.54	9.96	0.30	0.59	230.71	0.72
9	11/6/2008	-7.52	11.06	0.59	0.89	436.95	0.67
10	10/27/2008	-2.20	3.61	0.08	0.14	1603.41	0.50
11	12/2/2008	-4.96	9.41	0.30	0.64	701.32	0.70
12	6/13/2013	-	-	-	-	-	-
13	3/14/2011	-3.76	7.89	0.12	0.25	5900.01	0.67
14	1/22/2008	-4.07	9.25	0.28	0.71	368.56	0.68
15	12/12/2008	-47.25	52.83	3.20	3.60	298.49	0.72
16	8/17/2007	-10.67	22.86	0.26	0.59	30.25	0.99
17	11/13/2008	-4.95	9.80	0.34	0.73	288.28	0.74
18	5/30/2013	-	-	-	-	-	-
19	10/31/2008	-6.06	8.95	0.50	0.79	232.34	0.55
20	9/16/2008	-6.36	11.44	0.35	0.70	1630.85	0.74
21	1/15/2009	-4.92	10.07	0.33	0.73	183.70	0.75

In the first five columns, the crash date, average values of the parameters β_0, β_1 and the standard errors of those parameters estimated by the inverse of observed information matrix are shown for special quotes in crashes indicated in each of the days presented in Table 1 in the body text. The sixth and seventh columns show average values of Akaike's information criterion (AIC) and McFadden's R^2_{MC} that are defined by the expressions $AIC = -2(\log(L(\beta_0, \beta_1) - 2)$ and $R^2_{MC} = 1 - \log(L(\beta_0, \beta_1)/\log(L_0)$ respectively, where $L(\beta_0, \beta_1)$ is the likelihood function for the logistic regression being estimated and L_0 is one for null model $\beta_0 = 0$. There is no special quote in which the price was renewed more than four times on the days 5/23/2013, 6/13/2013, nor 5/30/2013.

Table 2: **Average values of parameters estimated using maximum-likelihood method for special quotes in rebounds.**

	Date	β_0	β_1	Std. error of β_0	Std. error of β_1	AIC	$R2\ MC$
1	10/14/2008	-8.95	14.41	0.50	0.80	2296.43	0.63
2	10/30/2008	-4.68	7.94	0.23	0.46	2751.59	0.58
3	10/29/2008	-13.92	17.81	0.96	1.20	1320.87	0.62
4	10/28/2008	-7.64	9.36	1.68	2.10	150.34	0.30
5	11/4/2008	-6.34	10.38	0.40	0.70	768.84	0.69
6	11/10/2008	-12.26	16.91	1.22	1.67	259.92	0.73
7	3/16/2011	-4.22	6.96	0.13	0.24	1381.06	0.66
8	11/25/2008	-6.47	10.48	0.42	0.68	659.97	0.59
9	12/15/2008	-11.14	17.22	1.06	1.61	452.32	0.74
10	12/8/2008	-11.91	16.07	1.21	1.68	532.34	0.83
11	3/13/2009	-28.93	32.53	1.41	1.61	677.11	0.77

The quantity shown in each column is the same as in Table 1.
